# Different Binding Properties and Function of CXXC Zinc Finger Domains in Dnmt1 and Tet1

**DOI:** 10.1371/journal.pone.0016627

**Published:** 2011-02-02

**Authors:** Carina Frauer, Andrea Rottach, Daniela Meilinger, Sebastian Bultmann, Karin Fellinger, Stefan Hasenöder, Mengxi Wang, Weihua Qin, Johannes Söding, Fabio Spada, Heinrich Leonhardt

**Affiliations:** 1 Department of Biology II and Center for Integrated Protein Science Munich (CIPSM), Ludwig Maximilians University Munich, Planegg, Germany; 2 Gene Center Munich, Ludwig Maximilians University Munich, Munich, Germany.; Wellcome Trust Centre for Stem Cell Research, United Kingdom

## Abstract

Several mammalian proteins involved in chromatin and DNA modification contain CXXC zinc finger domains. We compared the structure and function of the CXXC domains in the DNA methyltransferase Dnmt1 and the methylcytosine dioxygenase Tet1. Sequence alignment showed that both CXXC domains have a very similar framework but differ in the central tip region. Based on the known structure of a similar MLL1 domain we developed homology models and designed expression constructs for the isolated CXXC domains of Dnmt1 and Tet1 accordingly. We show that the CXXC domain of Tet1 has no DNA binding activity and is dispensable for catalytic activity *in vivo*. In contrast, the CXXC domain of Dnmt1 selectively binds DNA substrates containing unmethylated CpG sites. Surprisingly, a Dnmt1 mutant construct lacking the CXXC domain formed covalent complexes with cytosine bases both *in vitro* and *in vivo* and rescued DNA methylation patterns in *dnmt1^−/−^* embryonic stem cells (ESCs) just as efficiently as wild type Dnmt1. Interestingly, neither wild type nor ΔCXXC Dnmt1 re-methylated imprinted CpG sites of the *H19a* promoter in *dnmt1^−/−^* ESCs, arguing against a role of the CXXC domain in restraining Dnmt1 methyltransferase activity on unmethylated CpG sites.

## Introduction

In mammals DNA methylation is restricted to cytosine residues and mainly involves CpG dinucleotides. CpG methylation is widespread across mammalian genomes, including gene bodies regardless of their transcriptional activity [1]–[4]. However, highly CpG-rich regions (CpG islands) are refractory to methylation and mostly coincide with promoters of constitutively active genes. The methylation state of other regulatory sequences with moderate to low CpG density, including promoters and enhancers, shows developmental and/or tissue-specific variations and positively correlates with a transcriptionally silent state [1], [3]–[8]. Dense methylation of repetitive sequences is also thought to maintain these elements in a silent state and thus contribute to genome stability [9]–[11]. In mammals cytosine methylation is catalyzed by a family of DNA methyltransferases (Dnmts) [12]. Dnmt3a and Dnmt3b establish methylation patterns during embryonic development of somatic as well as germ cell lineages and, consistently, show developmental stage and tissue specific expression patterns. In contrast, Dnmt1 is ubiquitous and generally the most abundant DNA methyltransferase in mammalian tissues, where it associates with the replication machinery and restores symmetrical methylation at hemimethylated CpG sites generated by the semi-conservative DNA replication process [13]. Thus, Dnmt1 maintains methylation patterns with high fidelity and is essential for embryonic development and genome integrity [9], [14], [15].

**Figure 1:**
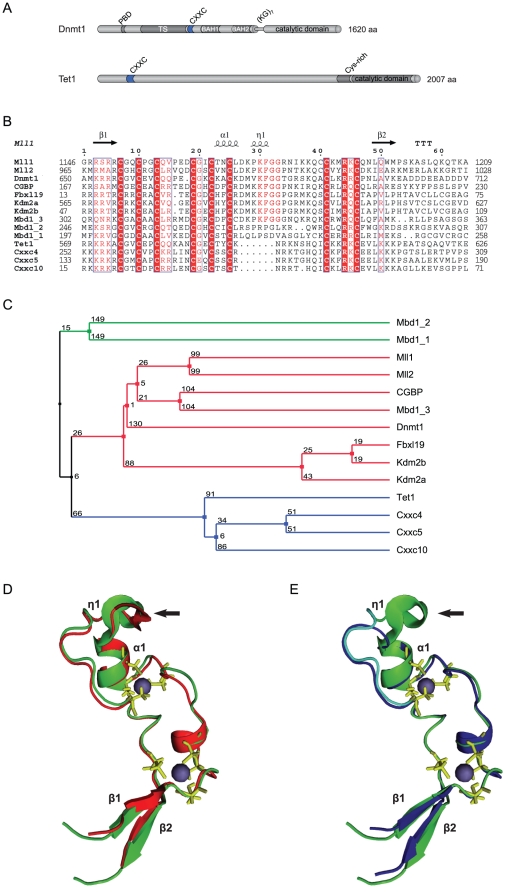
Sequence and predicted structural homology of CXXC domains. (**A**) Schematic representation of the domain structure in Dnmt1 and Tet1. The catalytic domain and the N-terminal region of Dnmt1 are connected by seven lysine-glycine repeats [(KG)_7_]. PBD: PCNA binding domain; TS: targeting sequence; CXXC: CXXC-type zinc finger domain; BAH1 and 2: bromo-adjacent homology domain; NLS: nuclear localization signal; Cys-rich: cysteine rich region. (**B**) Alignment of mammalian CXXC domains. Numbers on the right side indicate the position of the last amino acid in the corresponding protein. The Mbd1a isoform contains three CXXC motifs (Mbd1_1-3). Absolutely conserved residues, including the eight cysteines involved in zinc ion coordination are highlighted in red and the conserved KFGG motif is in red bold face. Positions with residues in red face share 70% similarity as calculated with the Risler algorithm [66]. At the top residues of MLL1 involved in β sheets β1 and β2 (black arrows), α helices α1 and α2 and strict α turns (TTT) are indicated. All sequences are from *M. musculus*. Accession numbers (for GenBank unless otherwise stated): Dnmt1, NP_034196; Mll1, NP_001074518; Mll4, O08550 (SwissProt); CGBP, NP_083144; Kdm2a, NP_001001984; Kdm2b, NP_001003953; Fbxl19, NP_766336; Mbd1, NP_038622; CXXC4/Idax, NP_001004367; CXXC5, NP_598448; CXXC10 (see Materials and Methods). (**C**) A homology tree was generated from the alignment in (B). The three subgroups of CXXC domains identified are in different colors. Average distances between the sequences are indicated. (**D–E**) Homology models of the mouse Dnmt1 (D; red) and Tet1 (E; blue) CXXC domains superimposed to the CXXC domain of MLL1 (green; [35]). MLL1 residues that were described to contact DNA according to chemical shift measurements [35] are cyan in (E), while cysteines involved in coordination of the two zinc ions are yellow. Arrows point to the KFGG motif in MLL1 and Dnmt1. The locations of α helices and β sheets are indicated as in (B).

Dnmt1 is a large enzyme with a complex domain structure that likely evolved by fusion of at least three genes [16]. It comprises a regulatory N-terminal region and a C-terminal catalytic domain connected by a linker of seven glycine-lysine repeats (Figure 1A)[17]. The N-terminal part contains a PCNA binding domain (PBD), a heterochromatin targeting sequence (TS), a CXXC-type zinc finger domain and two Bromo-Adjacent Homology domains (BAH1 and BAH2). The C-terminal domains of mammalian Dnmts contain all ten catalytic motifs identified in bacterial DNA (cytosine-5) methyltransferases [12]. Thus, prokaryotic and mammalian cytosine methyltransferases are thought to adopt the same catalytic mechanism. However, the C-terminal domain of Dnmt1 is the only DNA methyltransferase domain in Dnmts that is not catalytically active when expressed separately. Indeed, interaction with the N-terminal part is required for allosteric activation of the enzyme [18]. Remarkably, the first 580 amino acids (aa) of human DNMT1 are dispensable for both enzymatic activity and substrate recognition, whereas deletion of the first 672 aa results in an inactive enzyme [19]. Interestingly, this truncation eliminates part of the CXXC domain, suggesting an involvement of this domain in allosteric activation. However, addition of an N-terminal fragment containing the isolated CXXC domain to the catalytic domain was not sufficient for catalytic activation [20].

CXXC-type zinc finger domains are found in several other proteins with functions related to DNA or chromatin modification, including the histone H3 lysine 4 (H3K4) methyltransferases mixed-lineage leukaemia (MLL) proteins 1 and 4, the CpG-binding protein (CGBP, also known as Cfp1 or CXXC1), the methyl-CpG binding domain protein 1 (MBD1), the H3 lysine 36 (H3K36) demethylases KDM2A and B (also known as JHD1A/FBXL11 and JHD1B/FBXL10) and the MLL1 fusion partner TET1 (Figure 1A) [21]–[28]. The CXXC domains of some of these proteins were shown to mediate specific binding to double stranded DNA templates containing unmethylated CpG sites [21], [22], [29], [30]. A region of Dnmt1 which mainly includes the CXXC domain (aa 628–753) was also shown to bind Zn ions and DNA [20], [31], [32]. However, available data on the selectivity of this DNA binding activity are conflicting. Whereas a fragment including aa 613–748 of mouse Dnmt1 was shown to bind DNA with a slight preference for hemimethylated CpG sites [20], aa 645–737 of human DNMT1 were shown to selectively bind unmethylated DNA [32]. As these studies used different constructs and species, the selectivity of DNA binding by the CXXC domain of Dnmt1 with regard to CpG methylation state and the role of the CXXC domain in allosteric activation and substrate discrimination remain to be firmly established.

Notably, not all CXXC domains show DNA binding activity, as exemplified by the fact that only one out of three CXXC domains in MBD1 binds DNA [29]. Interestingly, TET1 was recently shown to be a 2 oxoglutarate- and Fe(II)-dependent dioxygenase responsible for converting genomic 5-methylcytosine (mC) to 5-hydroxymethylcytosine (hmC) [33], [34]. However, it is not known whether the CXXC domain of TET1 is involved in recognition of methylated DNA substrates.

Here we report a functional study and characterization of the DNA binding activity for the CXXC domains of mouse Dnmt1 and Tet1 proteins. We generated isolated CXXC domain and deletion constructs based on structural homology models to minimize structural alterations. We show that the CXXC domain of Dnmt1 preferentially binds DNA substrates containing unmethylated CpG sites, but does not contribute significantly to the DNA binding properties of the full length enzyme and is dispensable for its catalytic activity *in vitro* and *in vivo*. In addition, we found that the CXXC domain of Tet1 does not bind DNA *in vitro* and is also dispensable for catalytic activity of Tet1 *in vivo*.

## Results

### Sequence homology and structural modeling identify distinct CXXC domain subtypes

Dnmt1 contains a zinc finger domain of the CXXC type, which is present in several mammalian proteins including MLL1 (Figure 1A–C) and is highly conserved among Dnmt1 sequences from various animal species (Figure S1 in File S1). The primary structure of CXXC domains spans two clusters of 6 and 2 cysteine residues separated by a stretch of variable sequence and length. Sequence alignment and homology tree construction identified three distinct groups of CXXC domains (Figure 1B and C). The sequence between the two cysteine clusters in the CXXC domains of Dnmt1, CGBP/Cfp1, Fbxl19, Mll1, Mll2 and Kdm2 proteins and CXXC domain 3 of Mbd1 is highly conserved and contains a KFGG motif. The two other homology groups, including the CXXC domains 1 and 2 of Mbd1 on one side and those of Tet1, Cxxc4/Idax, Cxxc5/RINF and Cxxc10 on the other side, lack the KFGG motif and diverge from the first group and from each other in the sequence between the cysteine clusters. We generated structural homology models for the CXXC domains of mouse Dnmt1 and Tet1 using the NMR structure of the MLL1 CXXC domain as a template (Figure 1D and E)[35]. The CXXC domains of these proteins adopt an extended crescent-like structure that incorporates two Zn^2+^ ions each coordinated by four cysteine residues. The peptide of the MLL1 CXXC domain predicted to insert into the major groove of the DNA double helix (cyan in Fig. 1E) is located on one face of the structure and is contiguous to the KFGG motif [35]. The predicted structure of the Tet1 CXXC domain lacks the short 3_10_ helix (η1 in Figure 1E) formed by residues PKF and partially overlapping the KFGG motif, but is similar to the MLL1 CXXC domain in the region of the DNA-contacting peptide. However, each of the two predicted β-strands in Tet1 carries three positive charges, whereas there is only one or no charged residue in the C-terminal strands of the CXXC domains in MLL1 and Dnmt1. Depending on the orientation of the positively charged side chains, it cannot be excluded that the charge density prevents strand pairing in the Tet1 CXXC domain.

### The Dnmt1 CXXC domain binds unmethylated DNA

**Figure 2:**
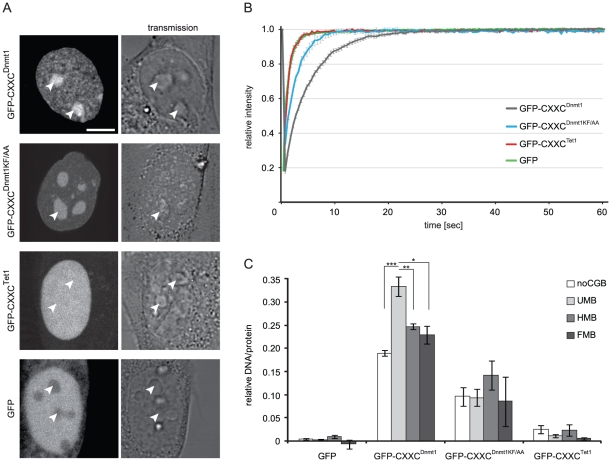
Properties of isolated Dnmt1 and Tet1 CXXC domains. (**A–B**) Subcellular localization (A) and binding kinetics (B) of GFP-CXXC^Dnmt1^, GFP-CXXC^Dnmt1KF/AA^, GFP-CXXC^Tet1^ and GFP in mouse C2C12 myoblasts. Localization and binding kinetics were independent from the cell cycle stage (Figures S2 and S5 in File S1). Arrowheads in (A) point to nucleoli. Scale bar: 5 µm. Binding kinetics were analyzed by FRAP. (**C**) DNA binding specificity of the Dnmt1 and Tet1 CXXC domains. GFP, GFP-CXXC^Dnmt1^, GFP-CXXC^Dnmt1KF/AA^ and GFP-CXXC^Tet1^ were pulled down from extracts of transiently transfected HEK293T cells and incubated with fluorescent DNA substrates containing no CpG site or one central un-, hemi- or fully methylated CpG site in direct competition (noCGB, UMB, HMB, FMB, respectively). Shown are the mean DNA/protein ratios and corresponding standard errors from 5 (GFP), 4 (GFP-CXXC^Dnmt1^ and GFP-CXXC^Dnmt1KF/AA^) and 2 (GFP-CXXC^Tet1^) independent experiments. * *P* = 0.01; ** *P* = 0.007; ***P = 0.001.

To investigate the binding properties of the Dnmt1 CXXC domain, we generated a GFP fusion construct including aa 652–699 (GFP-CXXC^Dnmt1^). According to our homology model the ends of this fragment form an antiparallel β-sheet that structurally delimits the domain as in MLL1. We first compared the localization and mobility of GFP-CXXC^Dnmt1^ and GFP in mouse C2C12 myoblasts. While GFP was diffusely distributed in both nucleus and cytoplasm, GFP-CXXC^Dnmt1^ was exclusively nuclear with a punctuated pattern throughout the nucleoplasm and was enriched in nucleoli, a pattern independent of cell cycle stage (Figure 2A and Figure S2 in File S1). Enrichment in the nucleus and nucleoli is frequently observed with constructs containing stretches with high density of basic residues. After photobleaching half of the nuclear volume we observed a slower fluorescence recovery rate for GFP-CXXC^Dnmt1^ than for GFP (Figure 2B). To rule out a contribution of nucleolar interactions to the slower kinetics of GFP-CXXC^Dnmt1^, we separately bleached nucleoplasmic and nucleolar regions and found that GFP-CXXC^Dnmt1^ has even faster kinetics within the nucleolus (Figure S3 in File S1). These results are consistent with a binding activity of GFP-CXXC^Dnmt1^ in the nucleus and very transient, unspecific binding in the nucleolus. To investigate whether the CXXC domain of Dnmt1 binds DNA and its possible selectivity with respect to CpG methylation we used a recently developed fluorescent DNA binding assay [36], [37]. GFP-CXXC^Dnmt1^ was transiently expressed in HEK293T cells, immunopurified with the GFP-trap (Figure S4 in File S1) and incubated with fluorescent DNA substrates containing either no CpG site or one central un-, hemi- or fully methylated CpG site in direct competition. As shown in Figure 2C, GFP-CXXC^Dnmt1^ displayed a significant preference for the substrate containing one unmethylated CpG site, which increased substantially with a five-fold higher concentration of the DNA substrates (Figure S5 in File S1). These results are consistent with the reported binding preference of the CXXC domains in human DNMT1 and other factors belonging to the same CXXC homology group [21], [22], [29], [32]. Notably, the CXXC domains 1 and 2 of Mbd1 lack the KFGG motif and do not bind DNA, while mutation of this motif prevented DNA binding by the CXXC domain of MLL1 [29], [38]. Therefore, we generated a GFP-CXXC^Dnmt1^ construct where the KFGG motif was mutated to AAGG (GFP-CXXC^Dnmt1KF/AA^, Figure S4 in File S1) to test the requirement of the KFGG motif for binding by the CXXC domain of Dnmt1. The mutant domain showed significantly decreased binding to all DNA substrates and complete loss of preferential binding to the unmethylated substrate *in vitro* (Figure 2B). In addition, GFP-CXXC^Dnmt1KF/AA^ showed faster recovery after photobleaching (FRAP) *in vivo* compared to the corresponding wild type construct (Figure 2C). These results further support the importance of the KFGG motif for DNA binding by CXXC domains.

### The CXXC domain of Tet1 shows no specific DNA binding activity and is dispensable for enzymatic activity *in vivo*

It was recently shown that Tet1 oxidizes genomic mC to hmC. However, the mechanism by which Tet1 is targeted to genomic mC is not known. Our model for the structure of the Tet1 CXXC domain diverged from the structure of the MLL1 CXXC domain with respect to the KFGG motif but not to the DNA-contacting peptide, suggesting that the Tet1 CXXC domain may still bind DNA. To test this we generated a GFP-tagged Tet1 CXXC construct (GFP-CXXC^Tet1^) following the same criteria as for GFP-CXXC^Dnmt1^ and investigated its cellular localization, *in vivo* binding kinetics and *in vitro* DNA binding activity. GFP-CXXC^Tet1^ was prevalently nuclear with a homogeneous distribution including nucleoli that was independent of cell cycle stage (Figure 2A and Figure S6 in File S1). After photobleaching GFP-CXXC^Tet1^ showed very fast recovery kinetics similar to GFP (Figure 2B) and its DNA binding activity *in vitro* was also similar to the background levels of the GFP control (Figure 2C). We conclude that the isolated CXXC domain of Tet1 has no specific DNA binding activity. Together with the observation that the CXXC domains 1 and 2 of Mbd1 also lack the KFGG motif and do not bind DNA [29] and that mutation of this motif reduced DNA binding by the CXXC domains of both Dnmt1 (Figure 2C) and MLL1 [38], this result indicates that the KFGG motif is a major determinant for DNA binding by CXXC domains.

**Figure 3:**
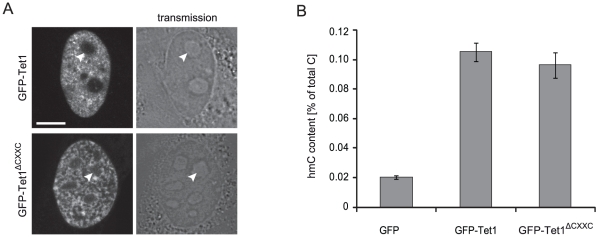
Cellular localization and *in vivo* catalytic activity of GFP-Tet1 and GFP-Tet1^ΔCXXC^. (A) Live images of C2C12 myoblasts expressing GFP-Tet1. Scale bar: 5 µm. (B) Genomic hmC content in HEK293T cells overexpressing GFP, GFP-Tet1 and GFP-Tet1^ΔCXXC^. Shown are mean values and standard deviation of hmC percentage over total cytosine for three measurements from one transfection.

To assess whether the CXXC domain is required for catalytic activity of Tet1 we generated a GFP-Tet1 fusion construct and a corresponding mutant lacking the CXXC domain (GFP-Tet1^ΔCXXC^). In C2C12 myoblasts GFP-Tet1 and GFP-Tet1^ΔCXXC^ showed punctuated nuclear patterns that did not depend on the cell cycle stage (Figure 3A and data not shown). The same constructs were transfected in HEK293T cells and global levels of genomic hmC were measured using a recently described hmC glucosylation assay [39]. Overexpression of GFP-Tet1 and GFP-Tet1^ΔCXXC^ determined a similar 5-fold increase of genomic hmC levels relative to control samples overexpressing GFP (Figure 3B), indicating that the CXXC domain is not required for enzymatic activity of Tet1 *in vivo*.

### Deletion of the CXXC domain does not affect the activity of Dnmt1 *in vitro*

**Figure 4:**
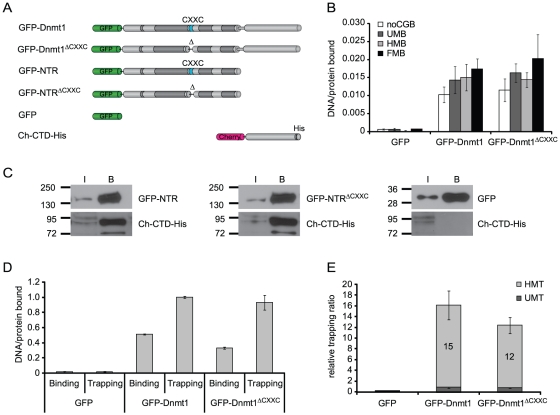
DNA binding specificity, intramolecular interaction and trapping of wild-type Dnmt1 and CXXC deletion constructs *in vitro*. (**A**) Schematic representation of Dnmt1 expression constructs. (**B**) DNA binding specificity of GFP-Dnmt1 and GFP-Dnmt1^ΔCXXC^ were assayed as described in Figure 2C. (**C**) Co-immunoprecipitation of the C-terminal domain of Dnmt1 (Ch-CTD-His) and the N-terminal region with and without deletion of the CXXC domain (GFP-NTR and GFP-NTR^ΔCXXC^, respectively). GFP fusions were detected using an anti-GFP antibody, while the C-terminal domain construct was detected using an anti-His antibody. GFP was used as negative control. I  =  input, B  =  bound. (**D**) Comparison of binding and trapping activities for GFP-Dnmt1 and GFP-Dnmt1^ΔCXXC^ to monitor irreversible covalent complex formation with hemimethylated substrates. (**E**) Relative covalent complex formation rate of GFP-Dnmt1 and GFP-Dnmt1^ΔCXXC^ on substrates containing one un- (UMT) or hemi-methylated CpG site (HMT) in direct competition. The trapping ratio for GFP-Dnmt1 on unmethylated substrate was set to 1. In (D) and (E) the means and corresponding standard deviations of triplicate samples from three independent experiments are shown. GFP was used as negative control.

To explore the role of the CXXC domain in Dnmt1 function we generated GFP-Dnmt1 fusion constructs where the CXXC domain, as defined by our homology model, was deleted. We reasoned that precise deletion of the entire structure delimited by the antiparallel β-sheet (Figure 1D) would have the highest chances to preserve native folding of the rest of the protein. We introduced this deletion in GFP fusion constructs encoding either the full length Dnmt1 or the isolated N-terminal region (GFP-Dnmt1^ΔCXXC^ and GFP-NTR^ΔCXXC^, respectively; Figure 4A and Figure S4 in File S1). We then compared DNA binding properties, catalytic activity and interaction between N-terminal region and C-terminal catalytic domain of ΔCXXC and corresponding wild type constructs. Competitive DNA binding assays with the same set of substrates as used for the experiments with GFP-CXXC^Dnmt1^ and GFP-CXXC^Tet1^ reported above (Figure 2C) showed that both GFP-Dnmt1 and GFP-Dnmt1^ΔCXXC^ bind DNA independently of the presence and methylation state of a CpG site (Figure 4B). As the isolated CXXC domain preferentially bound the substrate containing an unmethylated CpG site, the result with GFP-Dnmt1 and GFP-Dnmt1^ΔCXXC^ indicates that the CXXC domain contributes negligibly to the DNA binding specificity of the full-length enzyme.

Several groups reported that interaction between the N-terminal region and the C-terminal catalytic domain of Dnmt1 leads to allosteric activation of Dnmt1 [16], [18]–[20], [40]. To test whether the CXXC domain is involved in this intramolecular interaction, we co-expressed either GFP-tagged N-terminal region (GFP-NTR) or GFP-NTR^ΔCXXC^ constructs with a Cherry- and His-tagged C-terminal domain (Ch-CTD-His) in HEK293T cells and performed co-immunoprecipitation experiments. Ch-CTD-His co-precipitated both GFP-NTR and GFP-NTR^ΔCXXC^, indicating that the CXXC domain is dispensable for the interaction between the N-terminal region and the C-terminal domain of Dnmt1 (Figure 4C).

To investigate whether the CXXC domain is needed for enzymatic activity or substrate recognition, we tested formation of the covalent complex with cytosine and transfer of the methyl group for GFP-Dnmt1 and GFP-Dnmt1^ΔCXXC^. We first employed an assay to monitor covalent complex formation that exploits the formation of an irreversible covalent bond between the enzyme and the mechanism-based inhibitor 5-aza-2-deoxycytosine (5-aza-dC). This results in permanent trapping of the enzyme by DNA substrates containing 5-aza-dC, as opposed to the reversible complex formed with substrates containing the natural substrate 2-deoxycytosine (dC) [36]. GFP-Dnmt1 and GFP-Dnmt1^ΔCXXC^ were incubated with fluorescent DNA substrates containing either dC (binding) or 5-aza-dC (trapping) at a single CpG site in direct competition. DNA-protein complexes were then isolated by GFP pulldown and molar DNA/protein ratios were calculated from fluorescence measurements (Figure 4D). Covalent complex formation was then estimated by comparing trapping and binding activities. GFP-Dnmt1 and GFP-Dnmt1^ΔCXXC^ showed comparable covalent complex formation rates (trapping/binding ratios), which were about 15- and 12-fold higher for hemi- than un-methylated substrates, respectively (Figure 4E). Together with the data from binding experiments (Fig. 4B), this result indicates that the preference of Dnmt1 for hemimethylated substrates is determined at the covalent complex formation step rather than upon DNA binding. Furthermore, the CXXC domain clearly does not play a major role in determining either the efficiency or the methylation state-specificity of covalent complex formation.

Next, we tested whether deletion of the CXXC domain affects the ability of Dnmt1 to transfer [^3^H]methyl groups from the donor S-adenosylmethionine (SAM) to a poly(dI·dC)-poly(dI·dC) substrate, a standard DNA methyltransferase activity assay. This showed that *in vitro* GFP-Dnmt1 and GFP-Dnmt1^ΔCXXC^ are equally active methyltransferases (Figure S7 in File S1). This result is in contrast with a previous report showing that deletion of aa 647–690 in human DNMT1 encompassing the CXXC domain resulted in a drastic loss of catalytic activity [32]. However, according to our homology model the deletion by Pradhan *et al.* would eliminate the predicted N-terminal β-strand (β1 in Figure 1) preventing the formation of the antiparallel β-sheet and potentially distort the folding of the rest of the protein. This is in contrast with our GFP-Dnmt1^ΔCXXC^ mutant that was designed to retain the β-sheet structure. To test whether this may account for the observed discrepancy, we generated GFP fusion constructs of wild type human DNMT1 and the same deletion as reported by Pradhan *et al*. and tested covalent complex formation with 5-aza-dC containing DNA substrates as described above. While the human wild type construct showed the same preference for hemimethylated over unmethylated trapping substrates as the mouse constructs, this preference was clearly reduced for the human CXXC deletion mutant (Figure S8 in File S1). This result is consistent with the loss of enzymatic activity shown by Pradhan *et al*. for this mutant and together with the retention of trapping and catalytic activity by the different deletion in GFP-Dnmt1^ΔCXXC^ suggests that disruption of the antiparallel β-sheet delimiting the CXXC domain results in further distortion and loss of activity of the enzyme.

In conclusion, we showed that, *in vitro*, deletion of the CXXC domain does not affect the interaction between N-terminal region and C-terminal domain, DNA binding, the preference for hemimethylated substrates upon covalent complex formation and the methyltransferase activity of Dnmt1. Together, these data strongly argue against an involvement of the CXXC domain in allosteric activation of Dnmt1.

### Deletion of the CXXC domain does not affect Dnmt1 activity *in vivo*

**Figure 5:**
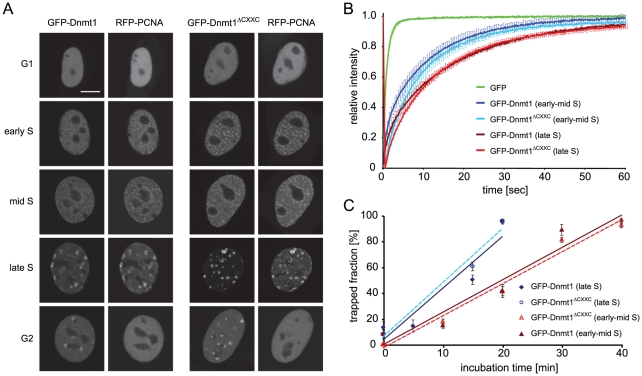
Cell cycle dependant cellular localization, protein mobility and trapping of wild-type Dnmt1 and CXXC deletion constructs in mouse C2C12 myoblasts. (**A**) Cell cycle dependent localization of GFP-Dnmt1 and GFP-Dnmt1^ΔCXXC^ constructs. Scale bar: 5 µm. (**B**) Analysis of binding kinetics of GFP-Dnmt1 and GFP-Dnmt1^ΔCXXC^ in early and late S-phase cells by FRAP. The recovery curve for GFP is shown for comparison. (**C**) *In vivo* trapping by FRAP analysis in cells treated with 5-aza-dC. The trapped enzyme fraction is plotted over time for early and late S-phase cells. For each construct three to six cells in early-mid and late S phase were analysed per time point. Shown are mean values ± SEM. In (A–C) RFP-PCNA was cotransfected to identify cell cycle stages in living cells.

We then undertook a functional characterization of the GFP-Dnmt1^ΔCXXC^ construct *in vivo*. We first compared localization and binding kinetics of GFP-Dnmt1 or GFP-Dnmt1^ΔCXXC^ in mouse C2C12 myoblasts co-transfected with RFP-PCNA, which served as S-phase marker [41]. GFP-Dnmt1^ΔCXXC^ showed the same cell-cycle dependent nuclear localization pattern as previously shown for GFP-Dnmt1 and endogenous Dnmt1 (Figure 5A)[42], [43]. Interaction with PCNA via the PBD directs Dnmt1 to replication foci throughout S-phase. In addition, in late S-phase and G2 Dnmt1 is enriched at chromocenters, clusters of pericentric heterochromatin (PH) that are observed as discrete domains densely stained by DNA dyes in mouse interphase cells. Association of Dnmt1 with PH at these stages is mediated by the TS domain [42]. Thus, the CXXC domain clearly does not contribute to the subnuclear localization of Dnmt1 at this level of resolution.

We also compared the mobility of GFP-Dnmt1 and GFP-Dnmt1^ΔCXXC^ in living C2C12 myoblasts by FRAP analysis (Figure 5B). These experiments revealed that the kinetics of Dnmt1 is not significantly affected by deletion of the CXXC domain in early-mid as well as late S-phase.

To test covalent complex formation in living cells, we used a previously established trapping assay [44]. Mouse C2C12 myoblasts were co-transfected with RFP-PCNA and either GFP-Dnmt1 or GFP-Dnmt1^ΔCXXC^ and treated with 5-aza-dC. Immobilization of the Dnmt1 constructs at the site of action was then measured by FRAP analysis (Figure 5C). GFP-Dnmt1 and GFP-Dnmt1^ΔCXXC^ showed very similar trapping kinetics, the immobile enzyme fraction reaching nearly 100% after 20 and 40 minutes in early-mid and late S-phase, respectively. This result clearly shows that the CXXC domain is dispensable for covalent complex formation also *in vivo*.

**Figure 6:**
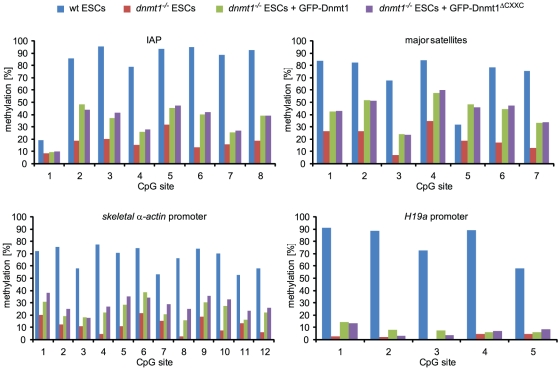
The CXXC deletion construct of Dnmt1 restores methylation in *dnmt1* null cells. Mouse *dnmt1^−/−^* ESCs transiently expressing GFP-Dnmt1 or GFP-Dnmt1^ΔCXXC^ were isolated by FACS-sorting 48 h after transfection and CpG methylation levels within the indicated sequences were analyzed by bisulfite treatment, PCR amplification and direct pyrosequencing. Methylation levels of untransfected wild type and *dnmt1^−/−^* ESCs are shown for comparison.

Finally, we compared the ability of GFP-Dnmt1 and GFP-Dnmt1^ΔCXXC^ to restore DNA methylation patterns in mouse *dnmt1^−/−^* ESCs. Cells transiently expressing either GFP-Dnmt1 or GFP-Dnmt1^ΔCXXC^ were FACS sorted 48 h after transfection. Isolated genomic DNA was then bisulfite treated and fragments corresponding to major satellite repeats, intracisternal type A particle (IAP) interspersed repeats, *skeletal α-actin* and *H19a* promoters were amplified and subjected to pyrosequencing (Figure 6). As shown previously [43], under these conditions GFP-Dnmt1 partially restored methylation of major satellite and IAP repeats and the *skeletal α-actin* promoter, but not of the imprinted *H19a* promoter, as establishment of the methylation imprint requires passage through the germ line [45]. Methylation patterns of all these sequences in cells expressing GFP-Dnmt1^ΔCXXC^ were very similar to those in GFP-Dnmt1 expressing cells, including the lack of (re-) methylation at the *H19a* promoter. These results suggest that the CXXC domain is not required for maintenance of DNA methylation patterns by Dnmt1 and does not restrain the DNA methyltransferase activity of Dnmt1 on unmethylated CpG sites. Thus, the CXXC domain does not play a major role in subcellular localization, it does not contribute to the global binding kinetics of Dnmt1 and, consistent with the *in vitro* data reported above, is dispensable for maintaining DNA methylation patterns in living cells.

## Discussion

We generated homology models based on the reported structure of the MLL1 CXXC domain to design isolated CXXC domain constructs and CXXC domain deletion mutants for Dnmt1 and Tet1 with minimal probability of structural alteration. According to these models CXXC domains are delimited by an antiparallel β-sheet, a discrete structural element. Our data show that the CXXC domain of mouse Dnmt1 preferentially binds DNA substrates containing unmethylated CpG sites as previously shown for CXXC domains of human DNMT1 and other mammalian proteins. We note that sequences C-terminal to the corresponding peptide in CGBP/Cfp1 were reported to be required for DNA binding *in vitro* [22] and that only a significantly larger peptide spanning the CXXC-3 domain of Mbd1a was tested for DNA binding. However, sequences C-terminal to CXXC domains are not conserved (Figure 1B) and our data show that they are not required for DNA binding by the CXXC domain of Dnmt1. Nevertheless, all the CXXC domains reported to selectively bind unmethylated CpG sites cluster in a distinct homology group and contain the KFGG motif. The latter was shown to be crucial for DNA binding by the CXXC domain of MLL1 [38] and here we extend this observation to the CXXC domain of Dnmt1. Sequence alignment reveals two distinct CXXC domain homology groups that lack the KFGG motif (Figure 1A). Consistent with a role of this motif in DNA binding, members of these groups such as CXXC-1/2 of Mbd1 [29] and the CXXC domain of Tet1 (this study) show no DNA binding activity. While no specific function is known for CXXC-1/2 of Mbd1, the CXXC domain of Tet1 is closely related to those in CXXC4/Idax and CXXC5/RINF that were shown to mediate protein-protein interactions [46]–[48]. This suggests that the CXXC domain of Tet1, rather than mediating DNA binding, may function as a protein-protein interaction domain. However, our data do not rule out the possibility that the DNA binding properties of the CXXC domain within the context of full length Tet1 may be different from those of the isolated domain. Nevertheless, we show that the CXXC domain is not required for enzymatic activity of Tet1 *in vivo*.

Although we observed a clear DNA binding activity by the isolated CXXC domain of Dnmt1, we found that, within the context of the full length enzyme, this domain is dispensable for overall DNA binding properties, preference for hemimethylated substrates upon covalent complex formation, methyltransferase activity and allosteric activation as well as for the ability to restore methylation of representative sequences in *dnmt1* null ESCs. Consistent with our data, a recent report showed a preference of the CXXC domain of human DNMT1 for substrates containing unmethylated CpG sites [32]. However, the same report showed that deletion of the CXXC domain from the human enzyme results in a significant decrease in methyltransferase activity on hemimethylated substrates *in vitro* and 25% lower methylation at rDNA repeats upon overexpression in HEK293 cells, suggesting a dominant negative effect of the deletion construct. These discrepancies may be due to the fact that the fragment deleted by Pradhan *et al.* includes the N-terminal strand of the predicted antiparallel β-sheet, potentially leading to disruption of native folding, to species-specific differences and/or to the analysis of non-physiological expression levels in HEK293 cells. In our trapping assay the same human deletion mutant showed reduced covalent complex formation, consistent with loss of enzymatic activity. The report from Pradhan *et al.* also showed that mutation of cysteine 667 to glycine within the CXXC domain of human DNMT1 disrupts DNA binding and enzymatic activity. However, as this point mutation involves one of the zinc coordinating residues it is not unlikely to alter peptide folding with negative consequences potentially extending beyond the CXXC domain and including reduced enzymatic activity. In this respect the dominant negative effect observed upon overexpression of this mutant may be explained by the prevalent occurrence of Dnmt1 as a dimer [49]. These observations, together with preserved ability for covalent complex formation and catalytic activity of our CXXC domain deletion, support the validity of our homology model-driven approach for functional characterization of the CXXC domain. In addition, our genetic complementation approach constitutes a rather physiologic functional assay. However, due to the transient approach and the analysis of genomic methylation at only a few representative sequences, subtle or highly sequence specific effects of deletion of the CXXC domain cannot be excluded.

It was recently shown that binding of Cfp1/CGBP and KDM2A to CpG islands through their CXXC domains leads to local enrichment and depletion of H3K4 and H3K36 methylation, respectively [26], [30]. Analogously, Dnmt1 may bind CpG islands through its CXXC domain. However, this interaction would not lead to a straightforward functional interpretation as CpG islands with high CpG density are generally refractive to DNA methylation and a function of Dnmt1 as a *de novo* DNA methyltransferase is not well established. It could be envisaged that binding to unmethylated CpG sites/islands by the CXXC domain may have a negative effect on the enzymatic activity of Dnmt1 and restrain its function as a *de novo* DNA methyltransferase. However, we show that in *dnmt1* null ESCs methylation of the imprinted *H19a* promoter is not restored upon expression of either wild type or ΔCXXC Dnmt1 constructs, arguing against a negative regulatory function of the CXXC domain.

Notably, binding of unmethylated CpG sites by KFGG motif-containing CXXC domains does not exclude a role in protein-protein interaction as the CXXC domain of MLL1 was reported to interact with both DNA and Polycomb Repressive Complex 1 components HPC2/CBX4 and BMI-1 [21], [50]. Therefore, it is possible that the CXXC domain of Dnmt1 has regulatory functions in specific cell types or developmental stages that may involve DNA binding and/or interaction with other proteins. The generation of dedicated animal models may be instrumental for testing these possibilities.

## Materials and Methods

### Bioinformatic methods

Alignments were performed using the ClustalW2 software [51]. The CXXC domain homology tree (Figure 1C) was generated from the alignment in Figure 1B with Jalview 2.4 by unweighted pair group method with arithmetic mean (UPGMA). The neighbor-joining method gave the same result. Average distances between the sequences were calculated using the BLOSSUM62 matrix. The human CXXC10 coding sequence [52] was determined by assembling ESTs AI438961, BX114363, BX492895, BU633058.1, AW207644.1 and the genomic sequence AC073046.7. The putative translational start site is located 16308 bp upstream of the annotated transcriptional start site of *TET3*. A partial coding sequence of murine Cxxc10 containing the CXXC domain was identified by aligning the human CXXC10 protein sequence to the ORFs present in NT_039353.7 upstream of the *tet3* gene from position 35663306 to 35808487). A very high match was found 13266 nt upstream of *tet3* at positions 35676374-35676572 of NT_039353.7. To build homology models for the CXXC domains of Dnmt1 (aa 645–696) and Tet1 (aa 561–614), we submitted the respective sequences to the HHpred server [53]. The best template was the CXXC domain of MLL1 (PDB-ID: 2J2S). The 49 residues of the CXXC domain in Dnmt1 can be aligned to this domain with 45% sequence identity and only a single amino acid gap after residue 661 (Figure 1B). 3D models were calculated with the homology modeling software MODELLER [54] (version 9.5) using this alignment. Distance restraints were given to MODELLER to enforce a distance of 2.3±0.1 Å between the eight sulphurs in the Zn-coordinating cysteines and the Zn^2+^ ions. TM-align [55] was used to superpose the model structure with the template domain. Images were generated using the PyMol Molecular Graphics System (Version 1.3, Schrödinger, LLC). The quality of the models and the underlying alignments were checked with DOPE [56] and Verify3D [57] and results for both models were found to be comparable to the MLL1 template structure (2J2S).

### Expression constructs

Fusion constructs were generated using enhanced green fluorescent protein, monomeric red fluorescent protein or monomeric cherry and are here referred to as GFP, RFP and Cherry fusions, respectively. Mammalian expression constructs for GFP, mouse GFP-Dnmt1, GFP-NTR and human RFP-PCNA were described previously [42], [44], [49], [58]. The deletion construct GFP-Dnmt1^ΔCXXC^ was obtained by replacing the sequence coding for aa 655–696 with three alanine codons in the GFP-Dnmt1 construct as described [59]. The GFP-DNMT1^ΔCXXC^ construct was generated by subcloning the sequence coding for human DNMT1^ΔCXXC^ from the homonymous construct by Pradhan *et al.* [32] in the pEGFP-C2 vector (Clonetech). To generate GFP-Tet1 three partially overlapping fragments spanning the Tet1 coding sequence were amplified using E14 ESCs cDNA as template. The fragments were then joined by overlap extension PCR and inserted into the pCAG-GFP-IB vector [43]. To generate GFP-Tet1^ΔCXXC^ aa 569-621 of murine Tet1 were deleted from GFP-Tet1 using a type IIs restriction endonuclease approach as described [60]. To generate GFP-CXXC^Dnmt1^ and GFP-CXXC^Tet1^ sequences coding for the respective CXXC domains (aa 643-700 for Dnmt1 and 561-614 for Tet1) were amplified by PCR using the GFP-Dnmt1 expression construct and cDNA from E14 ESCs as templates, respectively. PCR fragments were then inserted into the pCAG-GFP-IB vector. GFP-NTR^ΔCXXC^ was obtained by replacing the BglII-XhoI fragment of GFP-NTR with the same fragment of GFP-Dnmt1^ΔCXXC^. Ch-CTD-His was generated by replacing the GFP coding sequence in a GFP-CTD construct [49] with the Cherry coding sequence. All constructs were confirmed by sequencing.

### Cell culture, transfection and cell sorting

HEK293T cells [61] and mouse C2C12 myoblasts [62] were cultured in DMEM supplemented with 50 µg/ml gentamicin and 10% and 20% fetal calf serum, respectively. For expression of fusion proteins HEK293T cells were transfected with polyethylenimine (Sigma). For live cell imaging, C2C12 cells were grown to 40% confluence on Lab-Tek chambers (Nunc) or µ-slides (Ibidi) and transfected with TransFectin transfection reagent (BioRad) according to the manufacturer's instructions. Mouse ESCs were cultured as described [63] and transfected with FuGENE HD (Roche) according to the manufacturer's instructions. ESCs were sorted with a FACS Aria II instrument (Becton Dickinson). The *dnmt1^−/−^* J1 ESCs used in this study are homozygous for the c allele [14].

### *In vitro* DNA binding and trapping assays

*In vitro* DNA binding and trapping assays were performed as described previously [36], [37] with the following modifications. DNA substrates labeled with four different ATTO fluorophores (Tables S1 and S2 in File S1) were used at a final concentration of 125 nM each in the pull-down assay with immobilized GFP fusions. After removal of unbound substrate, the amounts of protein and DNA were determined by fluorescence intensity measurements with a Tecan Infinite M1000 plate reader using calibration curves from purified GFP or DNA coupled ATTO fluorophores, respectively. The following excitation/emission ± detection bandwidth settings were used: 490/511±10 nm for GFP, 550/580±15 nm for ATTO550, 600/630±15 nm for ATTO590, 650/670±10 nm for ATTO647N and 700/720±10 nm for ATTO700. Cross detection of GFP and different ATTO dyes was negligible with these settings. Binding and trapping ratios were calculated dividing the concentration of bound DNA substrate by the concentration of GFP fusion on the beads.

### *In vivo* mC hydroxylation assay

Genomic DNA was isolated from HEK293T cells 24 h after transfection with the GFP-Tet1 and GFP-Tet1^ΔCXXC^ constructs and global hmC levels were measured using the *in vitro* glucosylation assay as previously described [63], except that 100 nM β-glucosyltransferase and only UDP-[^3^H]glucose donor (0.43 µM) were used.

### Co-immunoprecipitation

Co-immunoprecipitation was performed as described previously [49], [64]. Shortly, HEK293T cells were transiently co-transfected with expression plasmids for GFP fusions and the Ch-CTD-His construct, harvested and lysed. GFP fusions were pulled down using the GFP-Trap [65] (Chromotek) and subjected to western blotting using anti-GFP (Roche or Chromotek) and anti-His (Invitrogen) monoclonal antibodies.

### Live cell microscopy, FRAP analysis and live cell trapping assay

Live cell imaging and FRAP experiments were performed as described previously [43]. For each construct 6-15 nuclei were averaged and the mean values as well as the standard errors were calculated. For presentation, we used linear contrast enhancement on entire images. The DNA methyltransferase trapping assay was described previously [44]. Briefly, transfected cells were incubated with 30 µM 5-aza-dC (Sigma) for the indicated periods of time before photobleaching experiments. FRAP analysis was performed with a confocal laser scanning microscope (TCS SP5, Leica) equipped with a 63×/1.4 NA Plan-Apochromat oil immersion objective. Microscope settings were as described except that a smaller region of interest (3 µm×3 µm) was selected for photobleaching. Mean fluorescence intensities of the bleached region were corrected for background and for total loss of nuclear fluorescence over the time course, and normalized by the mean of the last 10 prebleach values.

### DNA Methylation Analysis

Genomic DNA was isolated with the QIAmp DNA Mini Kit (Qiagen) and 1.5 µg were bisulfite converted using the EZ DNA Methylation-Gold Kit (Zymo research) according to the manufacturer's instructions. Primer sets and PCR conditions for IAP-LTR, *skeletal α-actin* and *H19* promoters were as described [43]. Primer sequences for major satellites were AAAATGAGAAACATCCACTTG (forward primer) and CCATGATTTTCAGTTTTCTT (reverse primer). For amplification we used Qiagen Hot Start Polymerase in 1x Qiagen Hot Start Polymerase buffer supplemented with 0.2 mM dNTPs, 0.2 µM forward primer, 0.2 µM reverse primer, 1.3 mM betaine (Sigma) and 60 mM tetramethylammonium-chloride (TMAC, Sigma). Promoter regions and IAP-LTR were amplified with two subsequent (nested) PCR reactions and major satellite repeats were amplified with a single amplification reaction. Pyrosequencing reactions were carried out by Varionostic GmbH (Ulm, Germany). Pyrosequencing primers are listed in Table S3 in File S1.

## Supporting Information

File S1 **Tables S1–S3, Figures S1–S8 and Supplemental methods.**
(PDF)
