# Supplementary material for: Different Binding Properties and Function of CXXC Zinc Finger Domains in Dnmt1 and Tet1
Source: PLoS One. 2011 Feb 2;6(2):e16627. doi: 10.1371/journal.pone.0016627 (PMC3032784; doi:10.1371/journal.pone.0016627)
Supplement: File S1 — Tables S1–S3, Figures S1–S8 and Supplemental methods. (PDF) [file pone.0016627.s001.pdf]

**Different binding properties and function of CXXC zinc finger domains in Dnmt1 and Tet1**

Carina Frauer[#], Andrea Rottach[#], Daniela Meilinger, Sebastian Bultmann, Karin Fellinger, Stefan Hasenöder, Mengxi Wang, Weihua Qin, Johannes Söding[§], Fabio Spada* and Heinrich Leonhardt*

Department of Biology and Center for Integrated Protein Science Munich (CIPS[M]), Ludwig Maximilians University Munich, Planegg-Martinsried and [§]Gene Center Munich, Ludwig Maximilians University Munich, Munich, Germany.

*Corresponding authors:
FS: f.spada@lmu.de; Fax +49 89 2180-74236; Tel. +49 89 2180-74230
HL: h.leonhardt@lmu.de; Fax +49 89 2180-74236; Tel. +49 89 2180-74232

[#]These authors contributed equally to this work

# SUPPLEMENTAL INFORMATION FILE

**Tables S1-3**

**Figures S1-8**

**Supplemental Methods**

**Table S1.** Sequences of DNA oligonucleotides used for preparation of double stranded DNA substrates. M: 5-methylcytosine.

| Name | Sequence |
|---|---|
| **CG-up** | 5’- CTCAACAACTAACTACCATCCGGACCAGAAGAGTCATCATGG -3’ |
| **MG-up** | 5’- CTCAACAACTAACTACCATCMGGACCAGAAGAGTCATCATGG -3’ |
| **noCG-up** | 5’- CTCAACAACTAACTACCATCTGGACCAGAAGAGTCATCATGG -3’ |
| **Fill-In-550** | 5’- ATTO550-CCATGATGACTCTTCTGGTC -3’ |
| **Fill-In-590** | 5’- ATTO590-CCATGATGACTCTTCTGGTC -3’ |
| **Fill-In-647N** | 5’- ATTO647N-CCATGATGACTCTTCTGGTC -3’ |
| **Fill-In-700** | 5’- ATTO700-CCATGATGACTCTTCTGGTC -3’ |

**Table S2**. DNA substrates used for the *in vitro* DNA binding and trapping assays.

| Name | CpG site | Label | Oligo I | Oligo II | dCTP reaction | Purpose |
|---|---|---|---|---|---|---|
| **noCGB 700** | no CpG site | 700 | noCG-up | Fill-In-700 | dCTP | Binding |
| **UMB 550** | | 550 | | Fill-In-550 | | |
| **UMB 590** | | 590 | | Fill-In-590 | dCTP | Binding |
| **UMB 647N** | unmethylated | 647N | CG-up | Fill-In-647N | | |
| **UMB 700** | | 700 | | Fill-In-700 | | |
| **UMT 550** | | 550 | | Fill-In-550 | 5-aza-dCTP | Trapping |
| **HMB 590** | | 590 | | Fill-In-590 | dCTP | Binding |
| **HMB 647N** | hemimethylated | 647N | MG-up | Fill-In-647N | | |
| **HMT 550** | | 550 | | Fill-In-550 | 5-aza-dCTP | Trapping |
| **HMT 647N** | | 647N | | Fill-In-647N | | |
| **FMB 647N** | fully methylated | 647N | MG-up | Fill-In-647N | 5methyl dCTP | Binding |

**Table S3**. Primers used for pyrosequencing. Each primer is biotinylated at the 5’ end.

| Name | Sequence |
|---|---|
| **skeletal α-actin-1** | 5’- AGTTGGGGATATTTTTTATA -3’ |
| **skeletal α-actin-1b** | 5’- TTTTGGTTAGTGTAGGAGAT -3’ |
| **skeletal α-actin-2** | 5’- TGGGAAGGGTAGTAATATTT -3’ |
| **H19-1** | 5’- ATAGTTATTGTTTATAGTTT -3’ |
| **H19-2** | 5’- AGGAATATGTTATATTTAT -3’ |
| **IAP LTR-1** | 5’- CCCTAATTAACTACAACCCA -3’ |
| **IAP LTR-2** | 5’- TGTAGTTAATTAGGGAGTGA -3’ |
| **Major Satellite-1** | 5’- AAAATGAGAAATATTTATTTG -3’ |
| **Major Satellite-2** | 5’- GAGAAATATATACTTTAGGA -3’ |

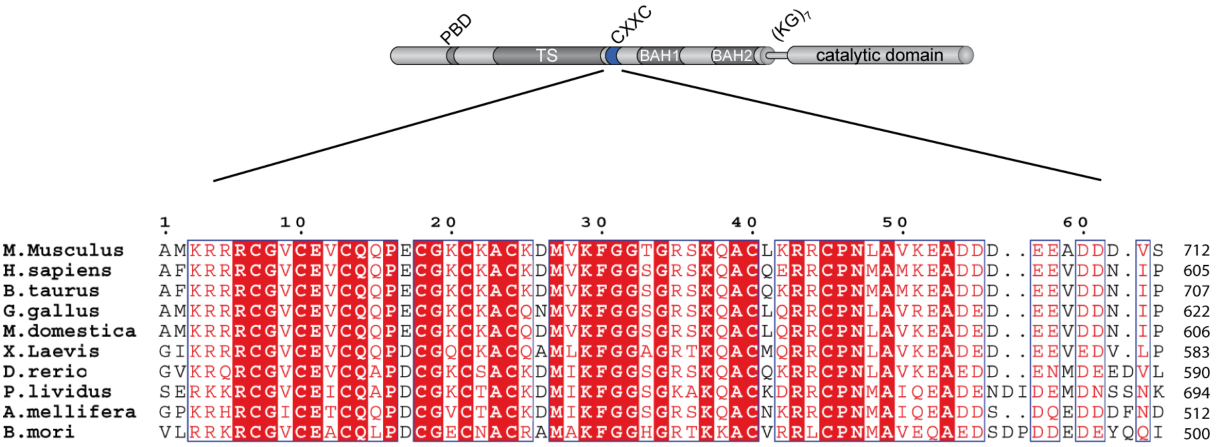


**Figure S1.** Dnmt1 domain structure and alignment of Dnmt1 CXXC domains from different species. Numbers on the right side indicate the position of the last amino acid in each sequence. PBD: PCNA binding domain; TS: targeting sequence; CXXC: CXXC-type zinc finger domain; BAH1 and 2: bromo-adjacent homology domain; (KG)7: seven lysine-glycine repeats. Absolutely conserved residues are highlighted in red. Positions with residues in red face share 70% similarity as calculated with the Risler algorithm {Mohseni-Zadeh, 2004 #133}. The alignment was generated with ClustalW2 and displayed with ESPript 2.2. GenBank accession numbers are: *Mus musculus*: NP_034196; *Homo sapiens*: NP_001124295; *Bos taurus*: NP_872592; *Monodelphis domestica*: NP_001028141; *Gallus gallus*: NP_996835; *Xenopus laevis*: NP_001084021; *Danio rerio*: NP_571264; *Paracentrotus lividus*: Q27746 (Swiss Prot); *Apis mellifera*: NP_001164522 (Dnmt1a); *Bombyx mori*: NP_001036980.

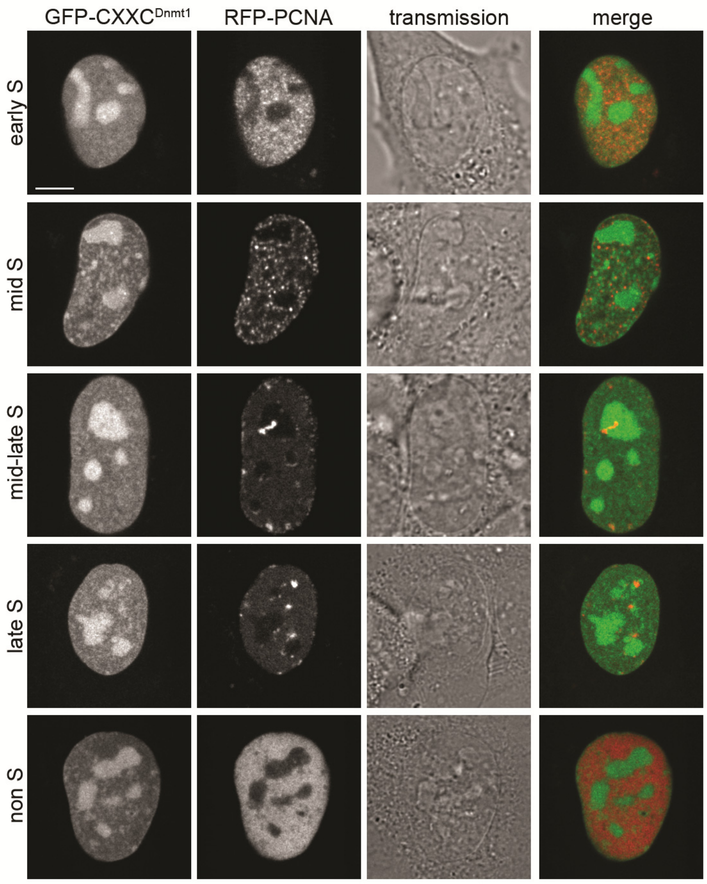


**Figure S2.** The cellular localization of GFP-CXXC$^{Dnmt1}$ is independent of cell cycle stage. Live images of C2C12 mouse myoblasts cotransfected with expression constructs for GFP-CXXC$^{Dnmt1}$ and RFP PCNA. The latter served for identification of the cell cycle stage. Scale bar: 5 µm.

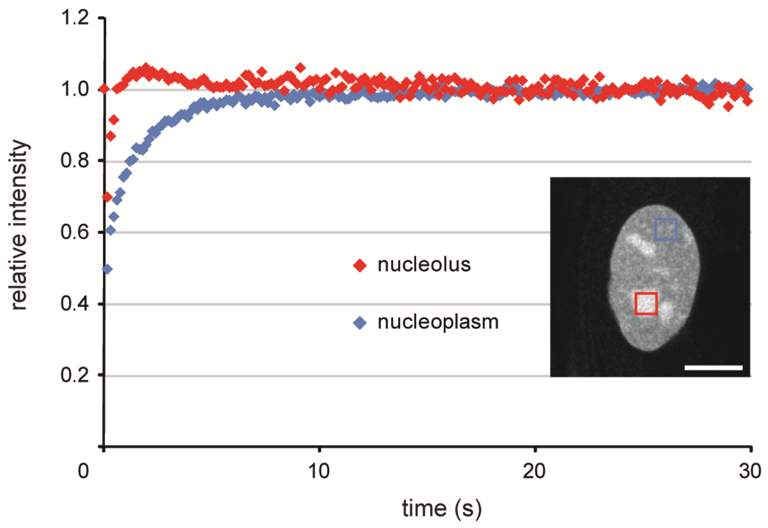


**Figure S3.** Differential mobility of GFP-CXXC$^{Dnmt1}$ in nucleoli and nucleoplasm of mouse C2C12 myoblasts measured by FRAP analysis. Identical regions of interest over the nucleoplasm or nucleoli (as exemplified in the inset) were bleached and recovery curves were recorded over 30 seconds. GFP-CXXC$^{Dnmt1}$ kinetics is faster in nucleoli than in the nucleus, which indicates more transient (possibly unspecific) binding in the former than in the latter. Scale bar: 5 µm.

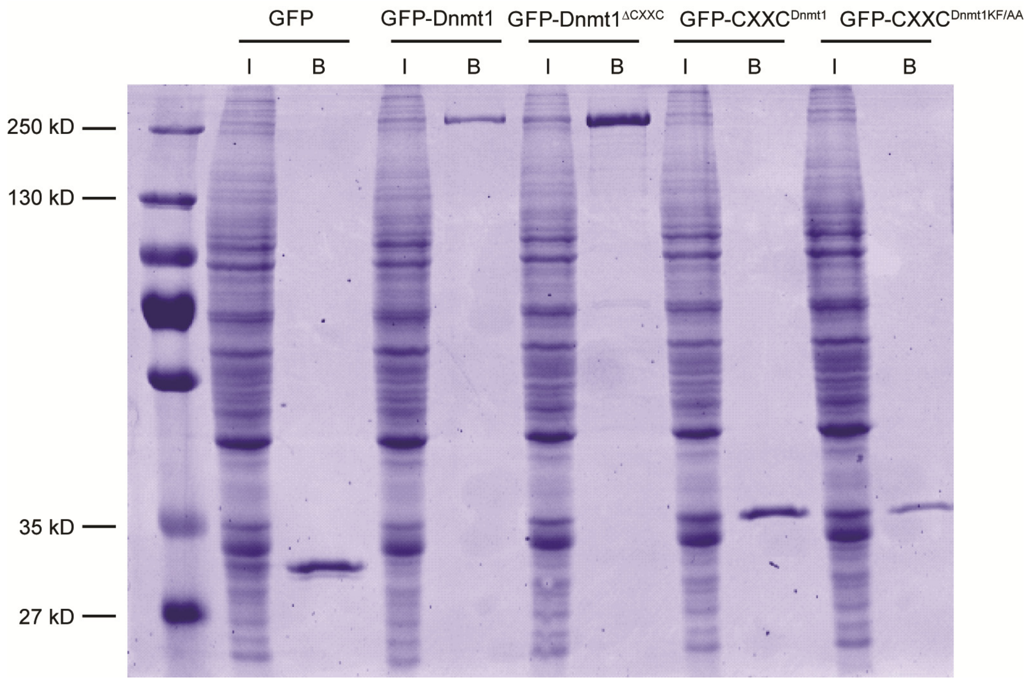


**Figure S4.** GFP fusion pulldowns from transiently transfected HEK293T cells using the GFP-trap. Shown is a SDS polyacrylamide gel stained with coomassie blue. I = input (1%); B = bound (10%).

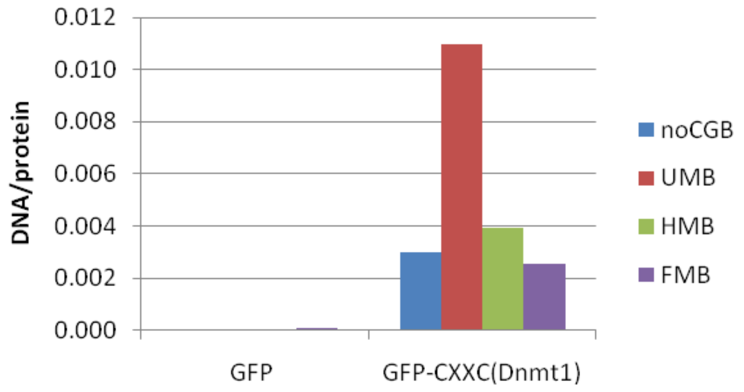


**Figure S5.** The CXXC domain of Dnmt1 preferentially binds unmethylated CpG sites. GFP and GFP-CXXC$^{Dnmt1}$ purified from transiently transfected HEK293T cells with the GFP trap were challenged with fluorescent DNA substrates containing no CpG site or one central un-, hemi- or fully methylated CpG site in direct competition (noCGB, UMB, HMB and FMB, respectively) as in Figure 2C, except that a five-fold higher concentration (625 nM) of each substrate was used.

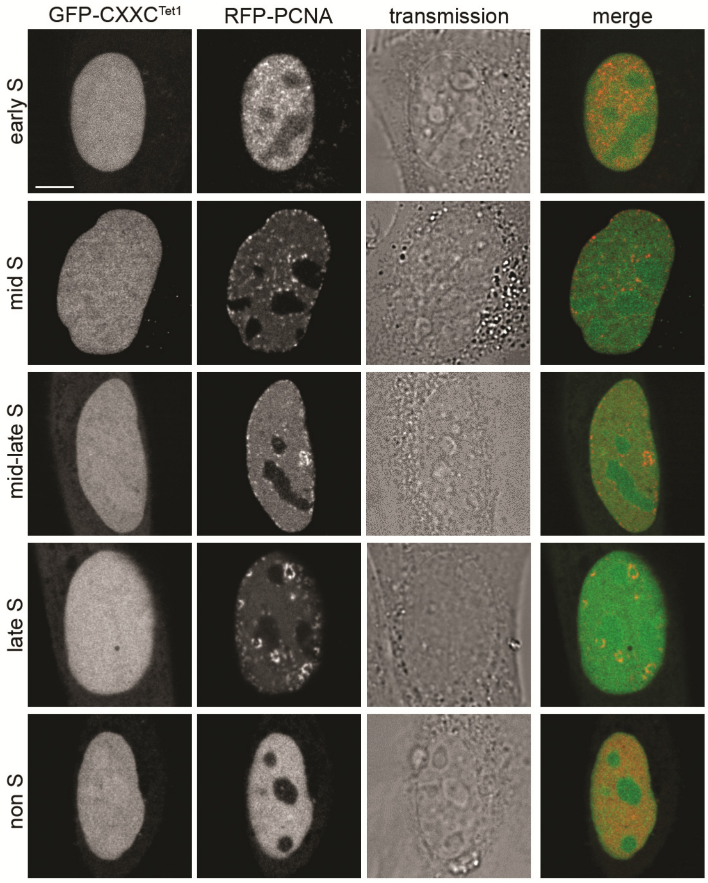


**Figure S6.** The cellular localization of GFP-CXXC$^{Tet1}$ is independent of cell cycle stage. Live images of C2C12 mouse myoblasts cotransfected with expression constructs for GFP-CXXC$^{Tet1}$ and RFP PCNA. The latter served for identification of the cell cycle stage. Scale bar: 5 µm.

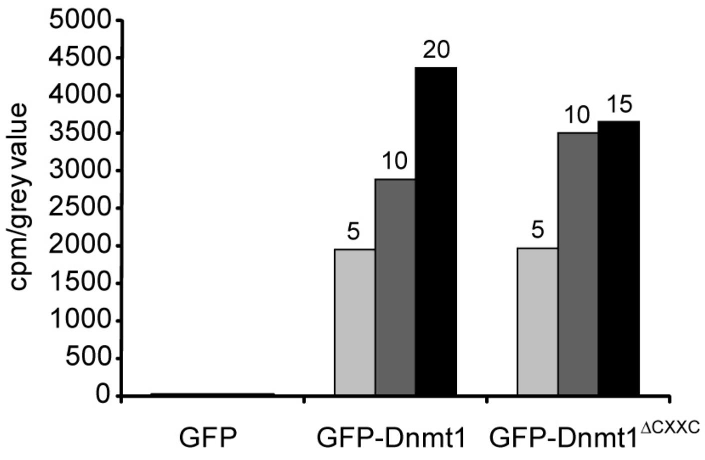


**Figure S7.** Radioactive methyltransferase activity assay for GFP Dnmt1 and GFP-Dnmt1$^{\Delta CXXC}$. The transfer of [3H]-methyl groups to poly(dI•dC)-poly(dI•dC) substrate was measured for increasing volumes of GFP fusion proteins immunopurified from transiently transfected HEK293T cells. Counts per minute (cpm) were normalized to the relative protein concentration as determined by SDS-PAGE analysis. GFP was used as negative control. Numbers above the bars indicate the volume (µl) of protein solution added.

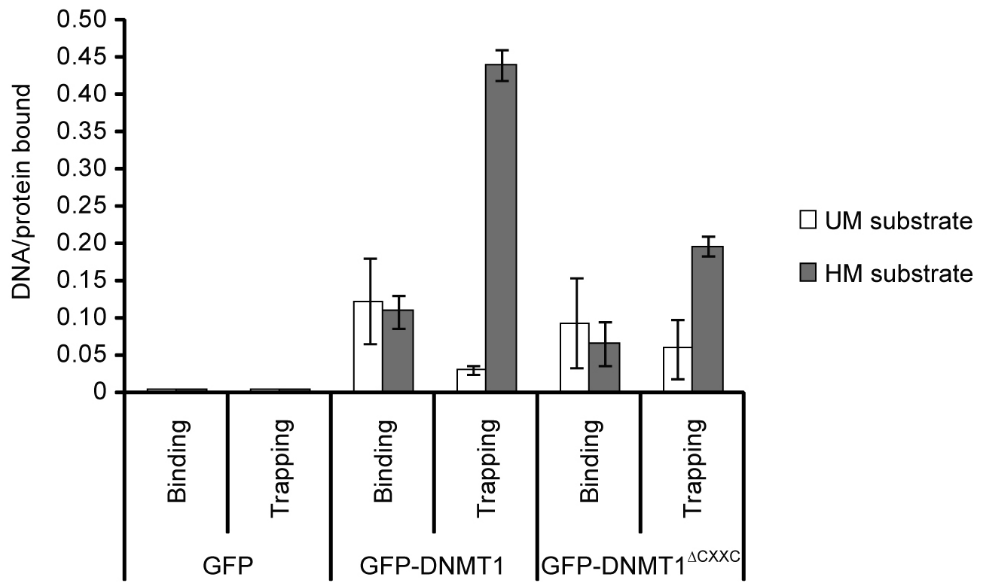


**Figure S8.** Competitive DNA binding and trapping assays for human GFP-DNMT1 and GFP-DNMT1$^{\Delta CXXC}$. GFP, GFP-DNMT1 and GFP-DNMT1$^{\Delta CXXC}$ were purified from transfected HEK293T cells using the GFP-trap and incubated with fluorescent DNA substrates containing one central unmethylated (UM) or hemimethylated (HM) CpG site in direct competition. Both substrates contained either dC (binding) or 5 aza dC (trapping) on the strand opposite to the differentially methylated one. The comparison of binding and trapping ratios reflects irreversible covalent complex formation. Note the reduction in trapping of GFP-DNMT1$^{\Delta CXXC}$ relative to GFP-DNMT1 by the hemimethylated substrate. Shown are mean values and standard deviation of DNA/protein ratios from two independent experiments.

**SUPPLEMENTARY METHODS**

***In vitro* methyltransferase activity assay**

Eight milligrams of His-tagged GFP-binding protein (GBP; Chromotek) were coupled to 1ml Ni-NTA agarose beads (Qiagen) by incubating for 2 h at 4°C in PBS and unbound protein was washed out twice with PBS. Extracts of HEK293T cells expressing GFP or a GFP fusions were prepared in 200 µl lysis buffer II (50 mM $NaH_2PO_4$ pH 8.0, 300 mM NaCl, 10 mM imidazole, 0.5 % Tween-20, 2 mM $MgCl_2$, 1 mg/ml DNaseI, 2 mM PMSF, 1X mammalian protease inhibitor mix). After centrifugation, supernatants were diluted to 500 µl with immunoprecipitation buffer II (50 mM $NaH_2PO_4$ pH 8.0, 300 mM NaCl, 10 mM imidazole, 0.05 % Tween-20) and precleared by incubation with 25 µl of equilibrated Ni-NTA agarose beads for 30 min at 4°C followed by centrifugation. Precleared extracts were then incubated with 40 µg of His-tagged GFP-trap coupled to Ni-NTA beads for 2 h at 4°C with constant mixing. GFP or GFP fusions were pulled down by centrifugation at 540 *g*. After washing twice with wash buffer II (50 mM $NaH_2PO_4$ pH 8.0, 300 mM NaCl, 20 mM imidazole, 0.05 % Tween-20), complexes were eluted with 60 µl of elution buffer (10 mM Tris pH 7.5, 100 mM KCl, 1 mM EDTA, 1 mM DTT, 250 mM imidazole) for 10 min at 25°C with constant mixing. 10 µl aliquots of all eluates were subjected to western blot analysis using mouse or rat monoclonal antibodies to GFP (Roche and Chromotek, respectively) and quantified by densitometry. Indicated volumes of eluate were incubated with 1 µg of poly(dI·dC)-poly(dI·dC) substrate (Sigma), 0.5 µg/µl of BSA and 1 µCi of S-adenosyl-[$^3$H-methyl]-methionine in 50 µl of trapping buffer (10 mM Tris pH 7.5, 100 mM KCl, 1 mM EDTA, 1 mM DTT) for 60 min at 37°C. 15 µl of each sample were spotted onto blotting paper and the DNA was precipitated with ice cold 5 % TCA. After washing twice with 5% TCA and once with cold 70 % ethanol, paper filters were air dried and analyzed by scintillation in 4 ml scintillation cocktail (Rotiszint® eco plus, Roth) for 5 min.
